# Severe Systemic Arthralgia After Zoledronic Acid Infusion in a Patient With Thyroid Dysfunction and Osteoporosis

**DOI:** 10.7759/cureus.83198

**Published:** 2025-04-29

**Authors:** Meumbur P Kpughur-Tule, Carly M Hubers, Kendall Conway, Reshma Reguram, Alexander M Satei, Durga Yerasuri

**Affiliations:** 1 Internal Medicine, Trinity Health Oakland, Pontiac, USA; 2 Internal Medicine, Wayne State University School of Medicine, Detroit, USA; 3 Diagnostic Radiology, Wayne State University, Detroit, USA; 4 Diagnostic Radiology, Trinity Health Oakland, Pontiac, USA; 5 Endocrinology, Trinity Health Livonia, Livonia, USA

**Keywords:** arthralgia, osteoporosis, severe allergic reaction, thyroid disease, zoledronic acid

## Abstract

Zoledronic acid (Reclast) is a commonly used bisphosphonate for the management of osteoporosis, but severe systemic inflammatory reactions following administration are uncommon. We report the case of a 72-year-old female who developed a novel case of severe arthralgia and systemic inflammatory symptoms following a single zoledronic acid infusion. This reaction occurred in the setting of newly diagnosed hyperthyroidism following prior hypothyroidism, uncontrolled hypertension, and osteoporosis. This case highlights the importance of recognizing severe systemic inflammatory reactions as a potential adverse effect of bisphosphonate therapy, particularly in patients with complex endocrine and metabolic comorbidities.

## Introduction

Zoledronic acid is widely prescribed for osteoporosis treatment due to its strong efficacy in reducing fracture risk. It is a third-generation nitrogen-containing bisphosphonate that inhibits osteoclast-mediated bone resorption by targeting the farnesyl pyrophosphate synthase enzyme in the mevalonate pathway, leading to osteoclast apoptosis and reduced bone turnover [1]. This potent antiresorptive property has made zoledronic acid a cornerstone in the management of osteoporosis and other metabolic bone diseases.

Since its approval by the FDA in 2001 for hypercalcemia of malignancy and later for osteoporosis in 2007, zoledronic acid has been widely used due to its ability to reduce fracture risk [2]. Clinically, zoledronic acid is indicated for the prevention and treatment of osteoporosis in postmenopausal women, glucocorticoid-induced osteoporosis, and osteoporosis in men. It is also used for the management of Paget’s disease, hypercalcemia of malignancy, and skeletal-related events in metastatic bone disease. Unlike oral bisphosphonates, zoledronic acid is administered annually as an intravenous infusion, enhancing compliance and providing sustained therapeutic benefits.

Common adverse effects include transient flu-like symptoms, fever, myalgia, and fatigue, which are typically mild and self-limiting [3]. However, more severe systemic inflammatory reactions, such as the one described in this case, are rare but clinically significant. We present a case of severe systemic inflammatory response with profound arthralgia following zoledronic acid infusion. The patient’s medical history included evolving thyroid dysfunction, uncontrolled hypertension, and osteoporosis. While the precise cause of her severe reaction remains uncertain, her underlying endocrine disorders may have contributed to the exaggerated inflammatory response. Given the rarity of such severe reactions, this case highlights the importance of recognizing systemic inflammatory responses as a potential complication of bisphosphonate therapy, particularly in patients with complex comorbidities.

## Case presentation

A 72-year-old female presented for evaluation of osteoporosis and hyperthyroidism. She had initially been on levothyroxine for hypothyroidism but developed persistently suppressed thyroid-stimulating hormone (TSH), measuring <0.01 μIU/mL on multiple occasions over the past year, and was later diagnosed with Graves’ disease, requiring methimazole treatment. Her thyroid-stimulating immunoglobulin (TSI) was negative, but anti-thyroid peroxidase (TPO) antibodies were significantly elevated at 494 IU/mL. Despite methimazole therapy, her free T3 remained elevated, with a peak of 10.3 pg/mL and recent levels of 5.0 pg/mL, while her free T4 was also intermittently elevated, reaching 3.13 ng/dL before showing improvement. A summary of relevant lab values can be found in Table 1.

**Table 1:** Laboratory findings Summary of key laboratory findings, including thyroid function tests and autoimmune markers with corresponding normal reference ranges. The table shows suppressed TSH, elevated thyroid hormones, and positive autoimmune markers, consistent with active thyroid dysfunction.

Laboratory Test	Result	Normal Range
TSH (μIU/mL)	<0.01	0.4 - 4.5
Free T3 (pg/mL)	5.0 (peak: 10.3)	2.3 - 4.2
Free T4 (ng/dL)	3.13	0.8 - 1.8
Anti-TPO Antibodies (IU/mL)	494	<35
Anti-Thyroglobulin Antibodies	Elevated	<4
24-hour Iodine Uptake (%)	34.6	10 - 30

She had a history of primary hypertension, managed with losartan and metoprolol, and had previously been evaluated for secondary causes of hypertension. An MRI of the adrenal glands did not demonstrate evidence of a pheochromocytoma. The patient also had a history of stage 3 chronic kidney disease, vitamin D deficiency, and erosive gastropathy.

**Figure 1:**
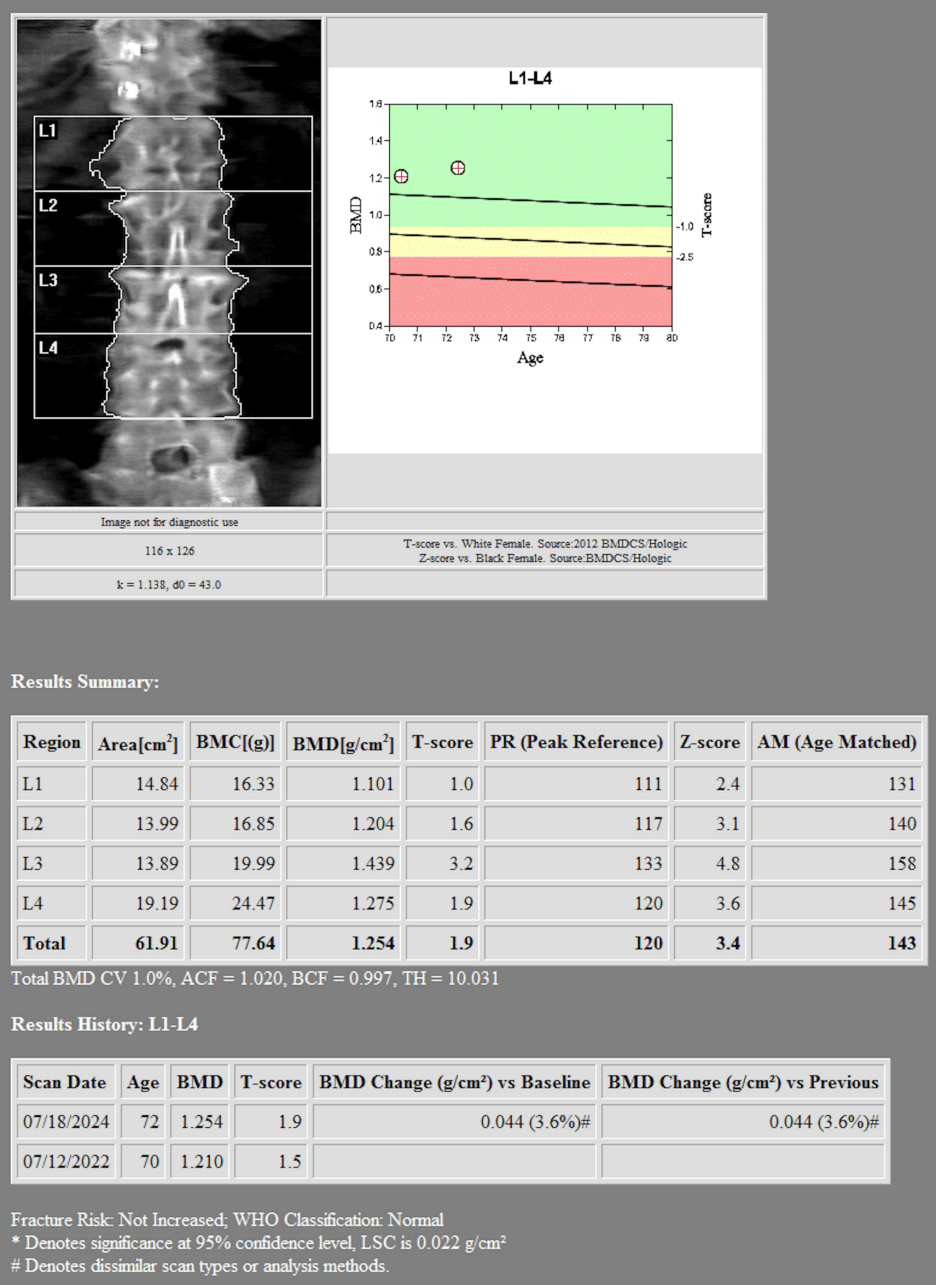
Lumbar spine DEXA DEXA scan showing lumbar spine bone mineral density of 1.254 g/cm² with a T-score of +1.9, indicating preserved bone mass at this site.

**Figure 2:**
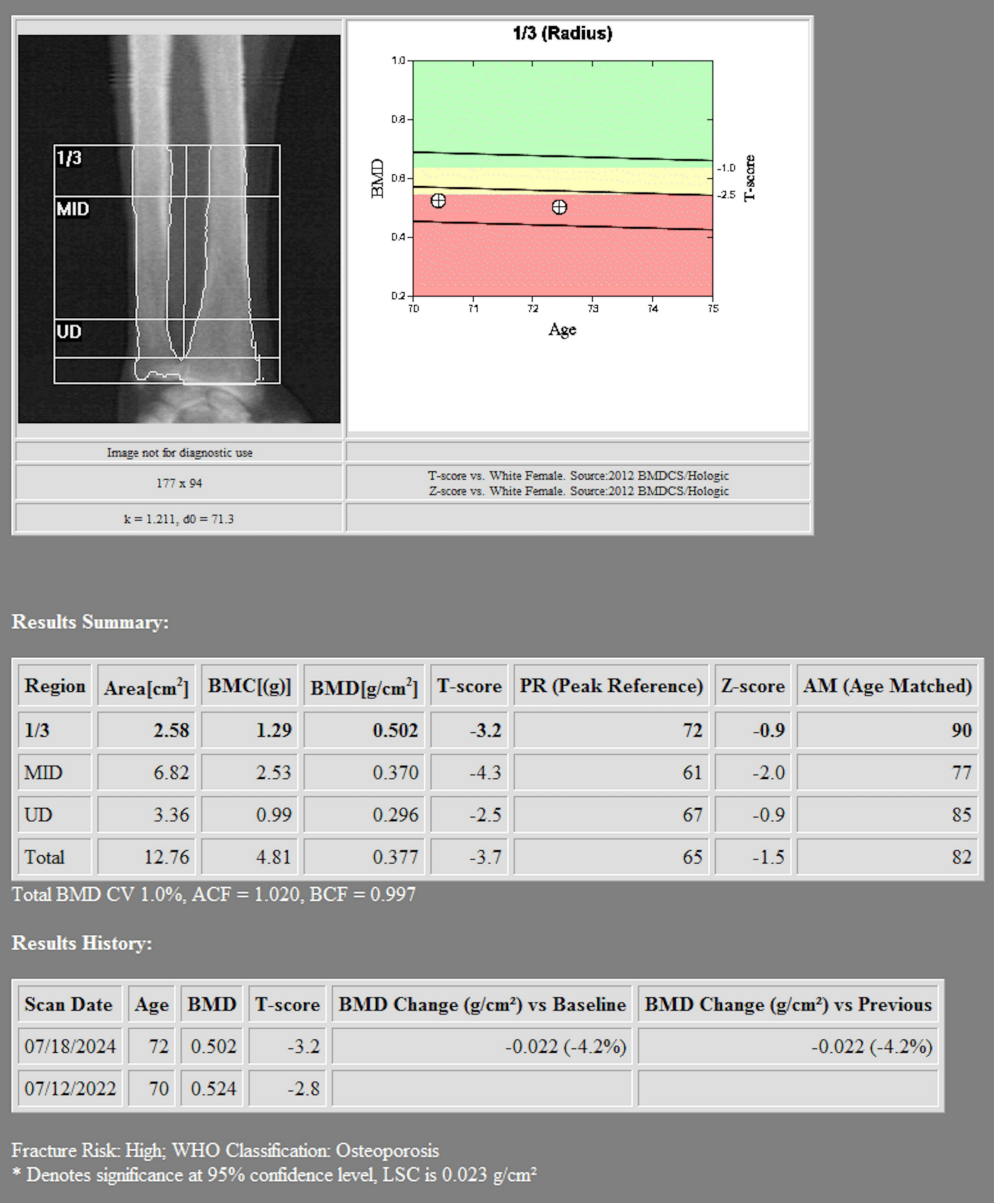
Radius DEXA Radius DEXA scan (33% measurement) demonstrating a bone mineral density of 0.502 g/cm² and a T-score of -3.2, consistent with significant osteoporosis.

**Figure 3:**
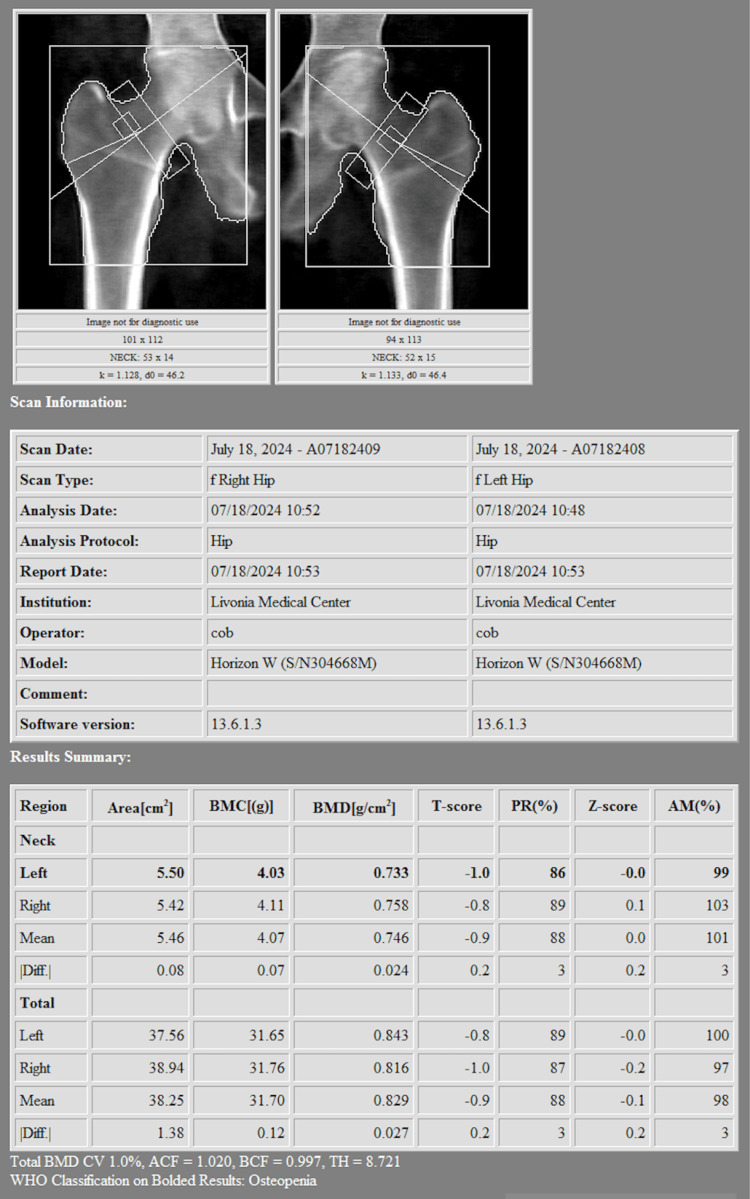
Femora DEXA Femora DEXA scan showing bone mineral densities of 0.733 g/cm² (left femoral neck) and 0.816 g/cm² (right femur), with T-scores of -1.0, consistent with osteopenia.

Her osteoporosis was confirmed with serial DEXA scans, which demonstrated a worsening trend in bone mineral density (BMD) over time. Her most recent dual-energy X-ray absorptiometry scan (DEXA) scan revealed a lumbar spine (L1-L4) BMD of 1.254 g/cm², with a T-score of +1.9, reflecting a +3.6% increase compared to the prior scan from two years prior (Figure 1). However, her right forearm (radius 33%) BMD showed a 4.2% decline, now at 0.502 g/cm², with a T-score of -3.2 indicating significant osteoporosis (Figure 2). The left femoral neck BMD decreased to 0.733 g/cm², with a T-score of -1.0, while the total right femoral BMD was 0.816 g/cm², with a T-score of -1.0, representing a 0.8% decline compared to the prior study (Figure 3). The WHO Fracture Risk Assessment Tool calculated a 3.6% risk of major osteoporotic fractures and a 0.4% risk of hip fractures, highlighting her ongoing fracture risk. Although the patient’s lumbar spine and femoral neck T-scores were in the osteopenic range, her radius T-score of -3.2 indicated significant cortical bone loss and elevated fracture risk. Given her stable stage 3 chronic kidney disease and absence of other contraindications, intravenous zoledronic acid was selected after careful consideration of the risks and benefits. The decision was made collaboratively between the endocrinology and primary care teams, with close post-infusion monitoring planned.

Given the complexity of the patient’s case, her thyroid dysfunction, hypertension, and osteoporosis were closely monitored. She received an intravenous infusion of zoledronic acid for osteoporosis management. The patient received the standard 5 mg intravenous dose of zoledronic acid, infused over at least 30 minutes per recommended guidelines. Given that her chronic kidney disease was stage 3 with stable renal function and no evidence of significant proteinuria or rapid progression, dose reduction was not performed.

Within a week of the infusion, she developed severe, diffuse arthralgia affecting her hands, wrists, knees, and lower extremities. Her symptoms were associated with significant leg swelling, muscle pain, and an increase in blood pressure beyond her baseline uncontrolled hypertension. The pain was severe enough to necessitate emergency care, where she was given analgesics. 

A summary of her key symptoms is provided here for clarity

1) Severe, diffuse arthralgia affecting the hands, wrists, knees, and lower extremities. 2) Significant bilateral leg swelling. 3) Muscle pain. 4) Marked increase in blood pressure above her baseline uncontrolled hypertension. 5) Need for emergency department evaluation due to symptom severity.

Diagnostic assessment

A comprehensive workup was later initiated to evaluate her adverse reaction to zoledronic acid. Laboratory evaluation after the infusion revealed persistent suppression of TSH (<0.01 μIU/mL) despite ongoing methimazole therapy, elevated free T3 levels (peak 10.3 pg/mL), and elevated anti-thyroglobulin antibodies. Vitamin D and B12 levels were within normal limits. These findings confirmed ongoing hyperthyroid activity with associated autoimmune markers.

**Figure 4:**
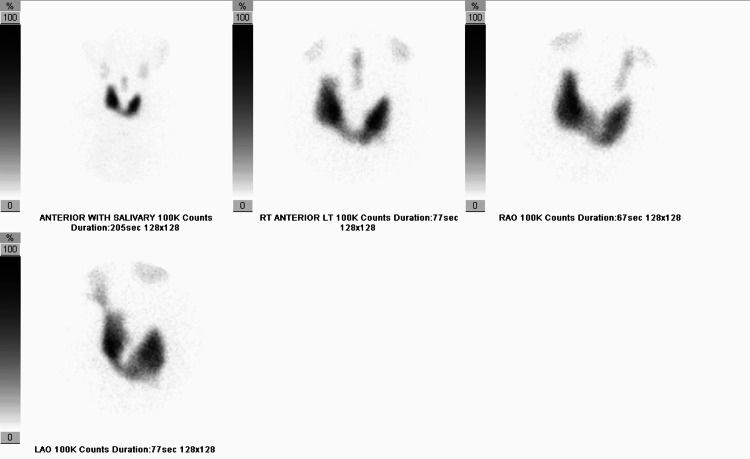
Thyroid scintigraphy Thyroid scintigraphy performed with I-131 sodium iodide and Tc-99m pertechnetate demonstrates homogeneously increased activity in the thyroid gland with an elevated 24-hour thyroid uptake value of 34.6%. No thyroid nodule was visualized. This is consistent with hyperthyroidism.

Thyroid scintigraphy demonstrated homogeneously increased radiotracer uptake within the thyroid gland, with a 24-hour iodine uptake of 34.6%, consistent with Graves' disease (Figure 4). A complete blood count and basic metabolic panel were largely within normal limits, aside from mild anemia. Inflammatory markers were not elevated. Overall, laboratory results suggested persistent hyperthyroid inflammation without evidence of a separate autoimmune or infectious process.

Treatment

The patient’s initial management in the emergency department included symptomatic treatment with acetaminophen and NSAIDs. Despite this, her symptoms persisted, and she followed up with her primary care physician, who prescribed a short course of prednisone. The corticosteroid therapy resulted in rapid and complete resolution of her symptoms.

Given the severity of her reaction, her primary care physician and endocrinologist advised against further bisphosphonate therapy. Zoledronic acid was subsequently added to her allergy list. Given her ongoing osteoporosis and the contraindication to bisphosphonates, alternative treatment strategies were discussed, including denosumab or selective estrogen receptor modulators (SERMs).

Outcome and follow-up

Following the resolution of her symptoms with prednisone, the patient did not experience a recurrence of severe arthralgia or systemic inflammatory symptoms. Her hypertension remained stable with continued losartan and metoprolol therapy. Thyroid function remained difficult to control, with persistently suppressed TSH despite methimazole therapy, necessitating continued endocrine follow-up.

Given her severe bisphosphonate reaction, she was advised against further zoledronic acid infusions. Instead, alternative osteoporosis management options, including denosumab, were considered. Non-pharmacologic measures such as adequate calcium and vitamin D supplementation and weight-bearing exercises were emphasized to optimize her bone health. At the time of this writing, the patient remains under close monitoring by her endocrinologist and primary care physician to assess long-term bone health and cardiovascular status.

## Discussion

Severe systemic inflammatory reactions following zoledronic acid infusion are rare, with few reported cases. A case report by Thangavelu et al. describes a rare but very severe systemic inflammatory response syndrome following zoledronic acid infusion, leading to multi-organ failure. The exact pathophysiology of this reaction remains unclear but may involve an exaggerated acute-phase response mediated by cytokine release. The current proposed mechanism involves excessive cytokine release, particularly TNF-α and IL-6, triggered by γδ T-cell activation [4]. While most bisphosphonate-induced acute phase reactions are mild with transient flu-like symptoms, this case highlights the potential for severe systemic responses. Our patient’s significant inflammatory reaction with severe arthralgia post-infusion aligns with this report, emphasizing the need for careful patient selection and monitoring. Further research is needed to identify predictors of severe bisphosphonate intolerance and guide individualized treatment.

Jamil et al. also describe a serious immune-mediated reaction following zoledronic acid infusion, where an elderly patient developed persistent joint pain, weakness, and significant functional impairment [5]. Similar to our case, symptoms emerged about a week post-infusion and progressively worsened rather than resolving within the expected 72-hour window of typical acute phase reactions. Notably, their patient exhibited an elevated erythrocyte sedimentation rate (ESR) despite negative autoimmune markers, suggesting an exaggerated inflammatory response rather than an underlying rheumatologic disease. Like our patient, she improved significantly with a prednisone taper, reinforcing the role of immune dysregulation in prolonged bisphosphonate reactions. This case further supports the need for heightened awareness of severe inflammatory responses to zoledronic acid, particularly in patients with predisposing autoimmune conditions or thyroid dysfunction.

However, our patient’s case remains unique due to several coexisting endocrine and metabolic conditions that may have influenced her response to zoledronic acid. Her hyperthyroid state could have exacerbated an inflammatory response, given the interplay between thyroid hormones and immune modulation [6]. Hyperthyroidism is linked to increased systemic inflammation, which may have amplified the patient’s immune response to bisphosphonate infusion [7]. Additionally, her history of uncontrolled hypertension raises questions about the role of vascular reactivity in bisphosphonate-induced systemic reactions. Studies have suggested that bisphosphonates can cause transient endothelial dysfunction, which may have contributed to her hypertensive crisis [8].

The patient’s MRI findings excluded a pheochromocytoma, which was in the differential as a potential contributor to her hypertensive episodes. However, her elevated TPO and anti-thyroglobulin antibodies suggest a complex autoimmune milieu that could predispose her to atypical drug reactions. Her chronic kidney disease and underlying vitamin D deficiency further complicate her osteoporosis management, as these conditions can alter calcium-phosphate homeostasis and bone remodeling [9].

DEXA imaging revealed osteoporosis due to the low T-score noted in the radius. These findings reinforce the importance of tailored osteoporosis treatment strategies, particularly for patients at risk of severe adverse reactions to bisphosphonates. Given the patient’s strong reaction, alternative treatments such as denosumab or SERMs should be considered. Additionally, nonpharmacologic measures, including adequate calcium and vitamin D intake and weight-bearing exercises, should be emphasized to optimize bone health [10].

Another consideration in this case is the role of systemic inflammation in worsening bone turnover and osteoporosis. The interplay between thyroid dysfunction and bone metabolism is well documented, with hyperthyroidism increasing bone resorption [11]. The patient’s history of thyroid dysfunction likely played a role in her ongoing osteoporosis and may necessitate closer monitoring for further bone loss.

## Conclusions

In conclusion, this case addresses the importance of recognizing that severe systemic inflammatory responses can occur after zoledronic acid infusion, particularly in patients with complex endocrine disorders. The patient's evolving thyroid dysfunction may have contributed to her exaggerated inflammatory reaction, suggesting that thyroid disease could be a risk factor for bisphosphonate intolerance. Clinicians should exercise caution when prescribing bisphosphonates in patients with significant endocrine comorbidities, including hyperthyroidism or autoimmune thyroid disease. Individualized risk assessment and careful monitoring are essential. Alternative treatments such as denosumab or SERMs should be considered for patients at higher risk of severe reactions. Future studies are needed to better understand the mechanisms behind severe bisphosphonate reactions and to identify predictive markers for safer osteoporosis management. Close collaboration between endocrinologists and primary care providers remains critical in managing patients with multifaceted metabolic and bone health concerns.
